# Applications of dynamic combinatorial chemistry for the determination of effective molarity†
†Electronic supplementary information (ESI) available: Derivation of equations used to determine of the effective molarities; experimental data relative to the determination of the equilibrium constants; ^1^H-NMR and ^13^C-NMR spectra of compound **1**. See DOI: 10.1039/c4sc02347a


**DOI:** 10.1039/c4sc02347a

**Published:** 2014-09-29

**Authors:** Maria Ciaccia, Irene Tosi, Laura Baldini, Roberta Cacciapaglia, Luigi Mandolini, Stefano Di Stefano, Christopher A. Hunter

**Affiliations:** a Dipartimento di Chimica and IMC/CNR , Università La Sapienza , P.le A. Moro 5 , 00185 Rome , Italy . Email: stefano.distefano@uniroma1.it; b Dipartimento di Chimica , Università di Parma , Parco Area delle Scienze 17/A , 43124 , Parma , Italy; c Department of Chemistry , University of Sheffield , Sheffield , S3 7HF , UK; d Department of Chemistry , University of Cambridge , Cambridge , CB2 1EW , UK . Email: herchelsmith.orgchem@ch.cam.ac.uk

## Abstract

A new strategy for determining thermodynamic effective molarities (EM) for macrocylisation reactions using dynamic combinatorial chemistry under dilute conditions is presented. At low concentrations, below the critical value, Dynamic Libraries (DLs) of bifunctional building blocks contain only cyclic species, so it is not possible to quantify the equilibria between linear and cyclic species. However, addition of a monofunctional chain stopper can be used to promote the formation of linear oligomers allowing measurement of EM for all cyclic species present in the DL. The effectiveness of this approach was demonstrated for DLs generated from mixtures of 1,3-diimine calix[4]arenes, linear diaminoalkanes and monoaminoalkanes. For macrocycles deriving from one bifunctional calixarene and one diamine, there is an alternating pattern of EM values with the number of methylene units in the diamine: odd numbers give significantly higher EMs than even numbers. For odd numbers of methylene units, the alkyl chain can adopt an extended all *anti* conformation, whereas for even numbers of methylene units, *gauche* conformations are required for cyclisation, and the associated strain reduces EM. The value of EM for the five-carbon linker indicates that this macrocycle is a strainless ring.

## Abstract 2

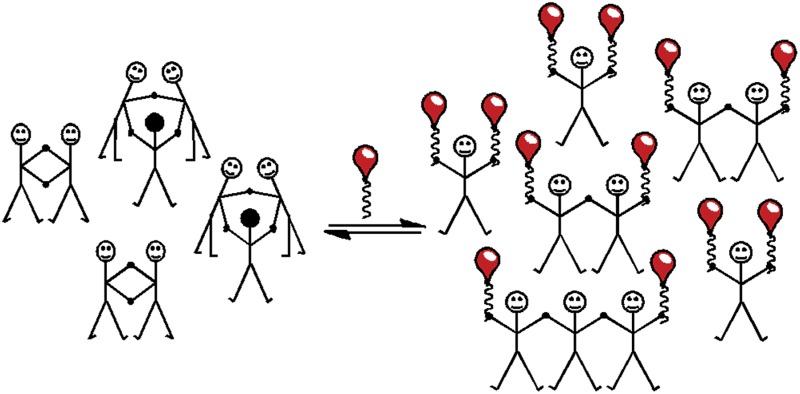
Chain-stoppers give rise to ring-chain equilibria in dynamic libraries allowing determination of thermodynamic effective molarities for macrocylisation reactions.

## Introduction

The growing thirst for *complexity* has fueled increasing attention on the study of dynamic systems in chemistry.^1^ Dynamic combinatorial chemistry^2^ represents one of the recent fields of supramolecular chemistry developed to generate complex systems under thermodynamic control. Libraries of interconverting compounds are generated by mixing suitable building blocks, and the distribution of species in such systems is determined by their relative stability and concentration.^3^ The diversity of the library is controlled by factors such as number of building blocks^4^ and the presence of templates.^5^ Concentration represents one of the key parameters determining the distribution of the species in a dynamic system generated from bifunctional building blocks. Substrates equipped with two functional groups, which are able to react with each other in a reversible fashion, produce a distribution of cyclic and linear oligomers that can be predicted using the theory of Jacobson and Stockmayer for macrocyclization under thermodynamic control.^3,6^ There is a critical value of the total concentration below which only the cyclic species form and above which the concentration of each macrocycle remains constant as the concentrations of the linear species increase with increasing total concentration. In other words, it is possible to generate “minimal dynamic libraries” of interconverting macrocycles by working in a range of concentration well below the critical value.

Such minimal libraries are still complex systems, as they contain many different combinations of molecular and supramolecular structures.^7^ The ease of formation of a particular macrocyclic species can be evaluated in terms of the effective molarity (𝔼𝕄), which is a quantitative measure of the relative efficiency of the intramolecular ring closure reaction of a linear bifunctional precursor and the corresponding intermolecular reaction between the same functional groups (eqn (1)).^3,8^ The effect of molecular structure on 𝔼𝕄 has been extensively investigated in both covalent^9^ and non-covalent systems.^10^ Since 𝔼𝕄 depends on both an entropic term associated with freezing free rotors and an enthalpic term associated with strain in the cyclic product, the nature of the linker between the reacting groups plays a crucial role in determining the ease of ring closure.1
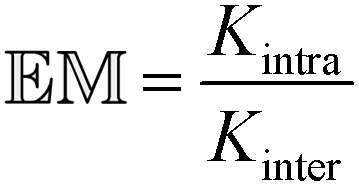
Herein we report the formation of minimal dynamic libraries of calix[4]arene macrocycles exploiting transimination reactions. Fine control of the composition is obtained by careful choice of the concentrations of starting materials and the length of the linker.

## Approach

**Fig. 1:**
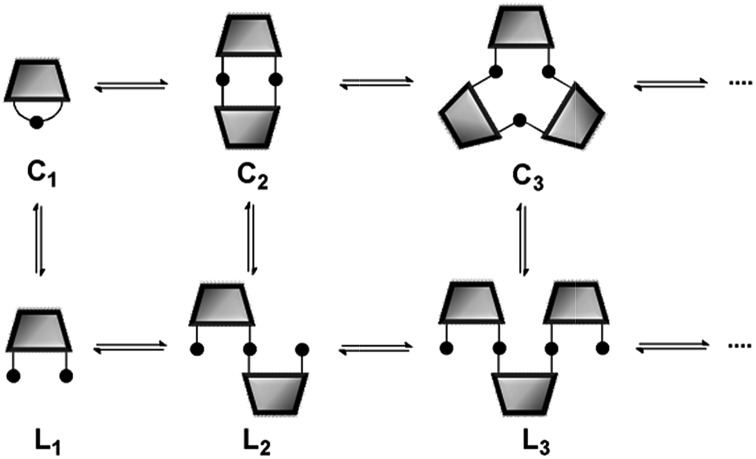
Schematic representation of a DL of bifunctional building blocks. **C_*n*_** are cyclic oligomers and **L_*n*_** are linear oligomers.

Experimental measurements of 𝔼𝕄 in dynamic libraries (DLs) of interconverting macrocycles have been previously reported.^3,4,5j,11^ The concentration of a macrocyclic *n*-mer, **C_*n*_**, is the product of the effective molarity for cyclisation, 𝔼𝕄_*n*_, and a factor which depends on the extent of the reaction.^12^ At low concentrations, only macrocyclic species are present, and the proportions of the different *n*-mers change with concentration. However, when the concentration increases above a certain threshold, linear oligomers, **L_*n*_**, are also present (Fig. 1). Under these conditions, the concentrations of the cyclic *n*-mers are independent of concentration, and the value of 𝔼𝕄_*n*_ is equal to the equilibrium concentration of **C_*n*_** in the DL.

**Fig. 2:**
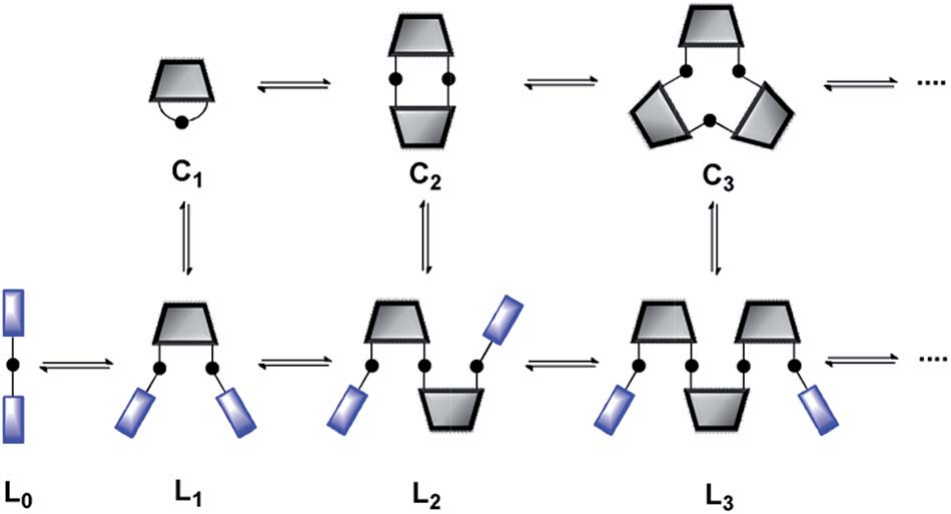
Schematic representation of a DL of monofunctional and bifunctional building blocks. **C_*n*_** are cyclic oligomers and **L_*n*_** are linear oligomers.

One disadvantage of this method is that effective molarities can only be measured at high concentrations, so solubility is an issue. Furthermore, in the case of expensive building blocks that are not available in bulk quantities, this approach is unsuitable. Here we report a new approach for the measurement of effective molarities in a DL under dilute conditions. The measurement of effective molarity in a DL requires the presence of both linear and cyclic species at equilibrium. A straightforward method for generating such a DL at low concentrations is to include monofunctional chain stoppers to promote the formation of open linear oligomers (Fig. 2).^13^

**Fig. 3:**
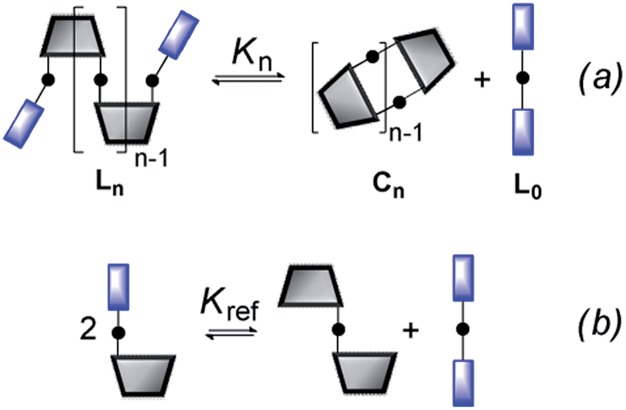
Schematic representation of the equilibria used to determine EM. (a) Cyclization of a linear species, **L_n_**, and (b) exchange reaction between monofunctional substrates.

The ratio of linear to cyclic species in the DL can be related to effective molarity by considering the equilibrium shown in Fig. 3a. If all the bonds connecting monomeric units in the oligomers have identical energies, then the equilibrium constant for the intermolecular reaction is equal to one and, consequently, the value of the microscopic EM_*n*_ is simply *K*_*n*_. multiplied by the symmetry number *σ*_*n*_ of the macrocycle.^14^ However, the two component system in Fig. 2 is more complicated than the one component system in Fig. 1, because there can be differences in the energies of the bonds formed between bifunctional and monofunctional building blocks. This difference is quantified by *K*_ref_, which must be measured in a control experiment using closely related monofunctional compounds (Fig. 3b). The value of microscopic EM_*n*_ is then given by eqn (2).2
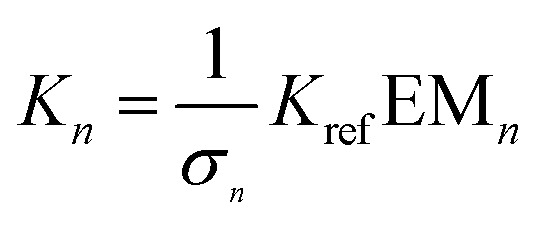



**Fig. 4:**
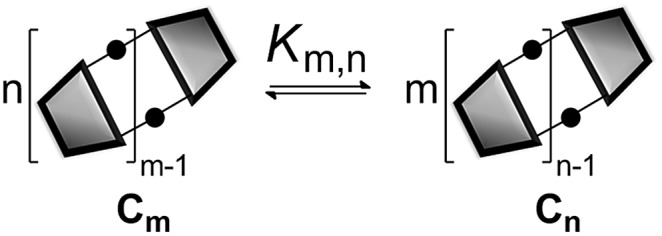
Schematic representation of the equilibrium used to determine the relationship between the effective molarities of two different macrocycles, EM_*n*_ and EM_*m*_.

In theory, measurement of the concentration of each macrocycle **C_*n*_** and the corresponding linear oligomer **L_*n*_** could be used to calculate all EM_*n*_ values for the system, but in practice, all of these species may not be present at detectable levels. However, once the effective molarity, EM_*m*_, of one macrocycle, **C_*m*_**, has been determined, the values of EM_*n*_ for all detectable cyclic species can be determined by considering the equilibrium depicted in Fig. 4 (eqn (3)).3
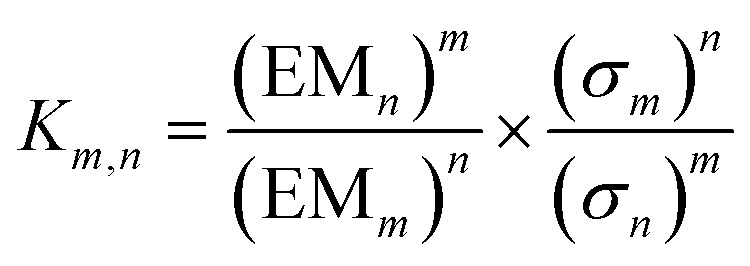
where *σ*_*m*_ and *σ*_*n*_ are the symmetry numbers of the two cyclic molecules.^8,15^

**Fig. 5:**
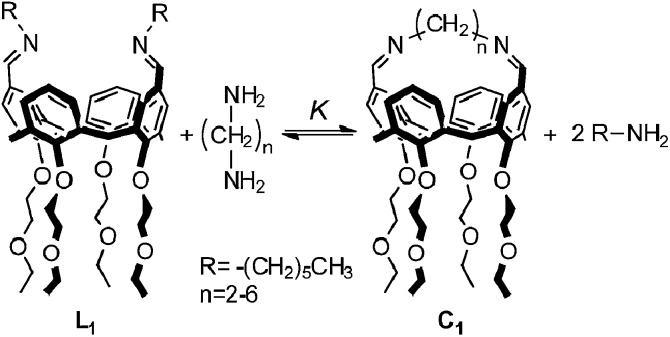
Equilibrium between the end-capped linear monomer **L_1_** and the cyclic monomer **C_1_**.

In this paper, we report the results obtained using this approach on dynamic libraries generated from a calixarene diimine and diamines. *Cone*-calix[4]arene derivatives were chosen as substrates due to their structural properties: the residual flexibility of a calix[4]arene locked in the *cone* conformation allows the approach of the distal positions favouring intramolecular reactions between suitably positioned functional groups.^16^ These compounds are therefore suitable platforms for the formation of cyclic species. Aliphatic diamines separated by an increasing number of methylenes were used to characterize the relationship between EM and chain length. Fig. 5 shows the chemical structures of the cyclic monomer, **C_1_**, and the end-capped linear monomer, **L_1_**, and represents the equilibrium depicted in Fig. 3a adapted to the specific case of a DL generated from two bifunctional building blocks and *n* = 1.

## Results

**Fig. 6:**
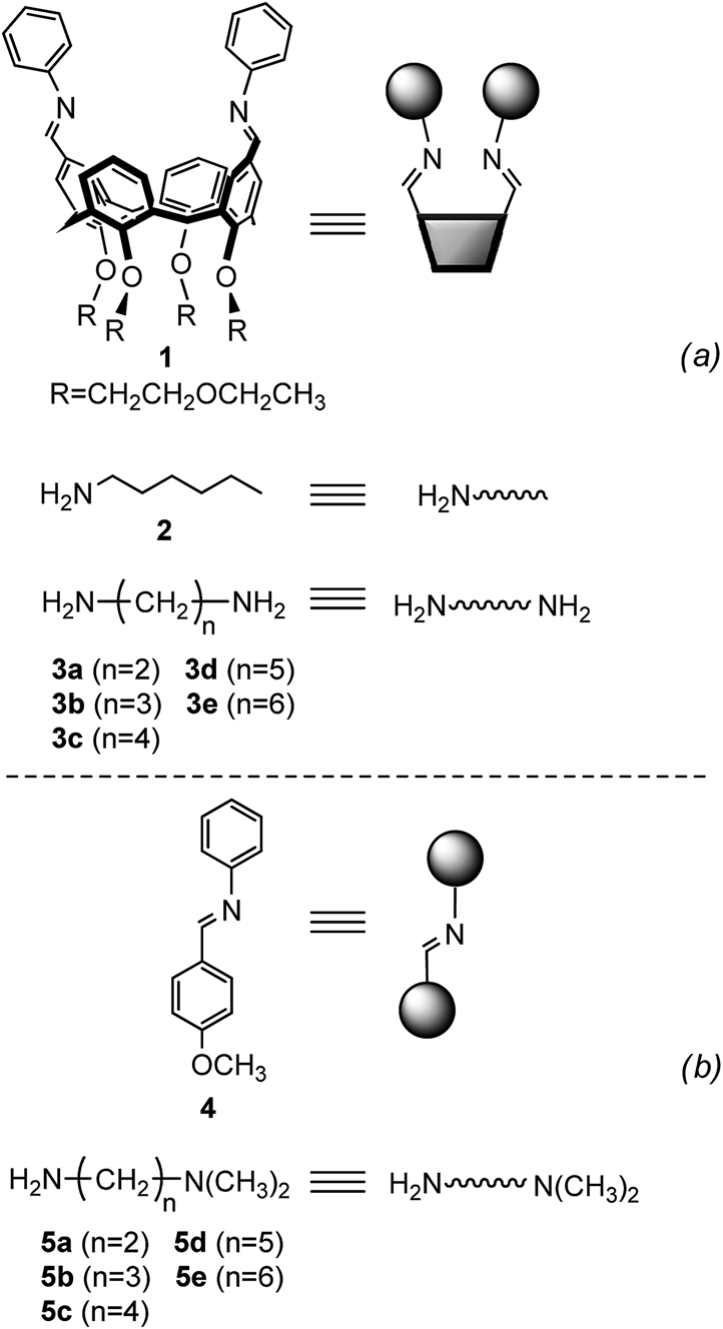
Building blocks used for the generation of the dynamic systems of equilibrating imines (a) and monofunctional substrates used for the reference reaction (b). Cartoons used to represent the building blocks are also shown.

**Fig. 7:**
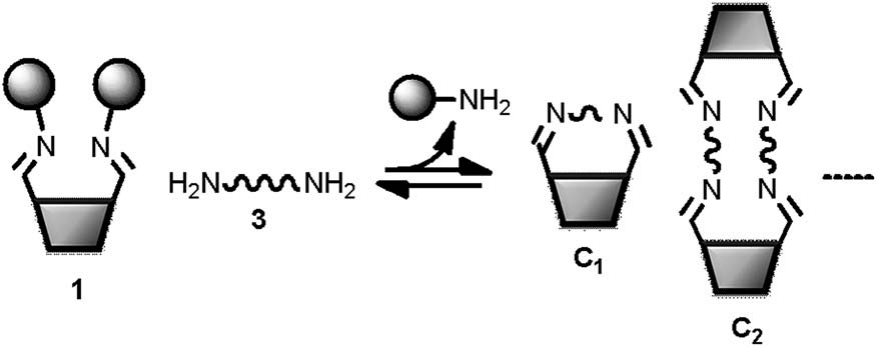
Schematic representation of a DL of cyclic imines generated from the bifunctional building blocks **1** and **3** (see Fig. 6 for key).

Minimal libraries of interconverting imines were generated in CD_3_CN starting from the building blocks represented in Fig. 6a. We have shown previously^17^ that in organic solvents transimination proceeds faster than the corresponding imine formation reaction in the absence of any Brønsted or Lewis acid catalyst. For this reason, the 1,3-distal diformylated *cone*-calix[4]arene was converted to the imine derivative **1**. The aniline moieties in **1** ensure that the transiminations with amines **2** and **3a–e** (Fig. 7) are biased toward the aliphatic imine products.^17*b*^

**Fig. 8:**
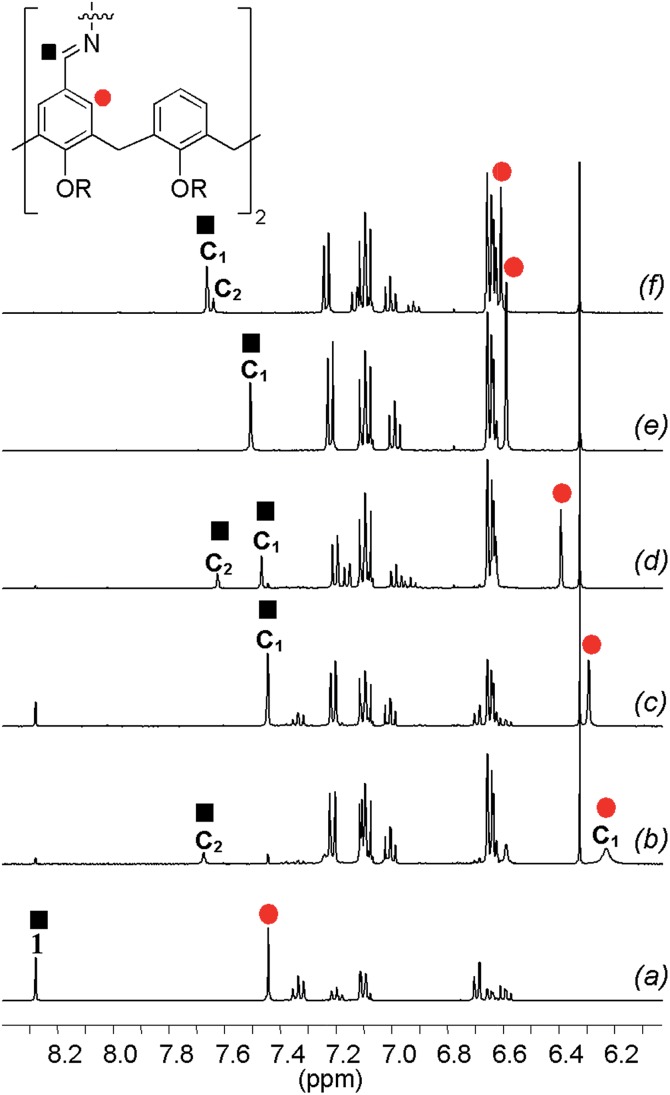
Aromatic region of the ^1^H-NMR spectra of **1** (a) and of equimolar equilibrated mixtures of **1** (2.5 mM) and each of the diamines **3a–e** (b–f) recorded in CD_3_CN at 298 K. The signal due to the imine proton of **C_1_** is not observed in (b) and is probably hidden under the aromatic signals between 6.5 and 7.4 ppm.

In order to characterize the system, the DLs generated from building blocks **1** and **3** (Fig. 7) were monitored by ^1^H-NMR spectroscopy in CD_3_CN. Since both linear and cyclic species can be formed from bifunctional building blocks, low concentrations of starting materials were used to favour the formation of cyclic species. In the spectra of the equilibrated mixtures^18^ either one or two new imine signals were observed. Fig. 8 shows partial ^1^H-NMR spectra of **1** and of mixtures of **1** and **3**. Both **C_1_** and **C_2_** (see characterization below) were observed in the DLs generated from diamines with an even number of methylenes (**3a**, **3c** and **3e** in Fig. 8b, d and f, respectively) whereas only **C_1_** was formed from **3b** and **3d**, which have an odd number of methylenes (Fig. 8c and e, respectively). The ^1^H NMR chemical shifts of the signals due to the cyclic species are at significantly higher fields than the corresponding signals of **1**. The signals due to the calixarene ring proton adjacent to the imine moiety (marked by the red circle in Fig. 8) are increasingly shielded as the length of the diamine chain decreases.

**Fig. 9:**
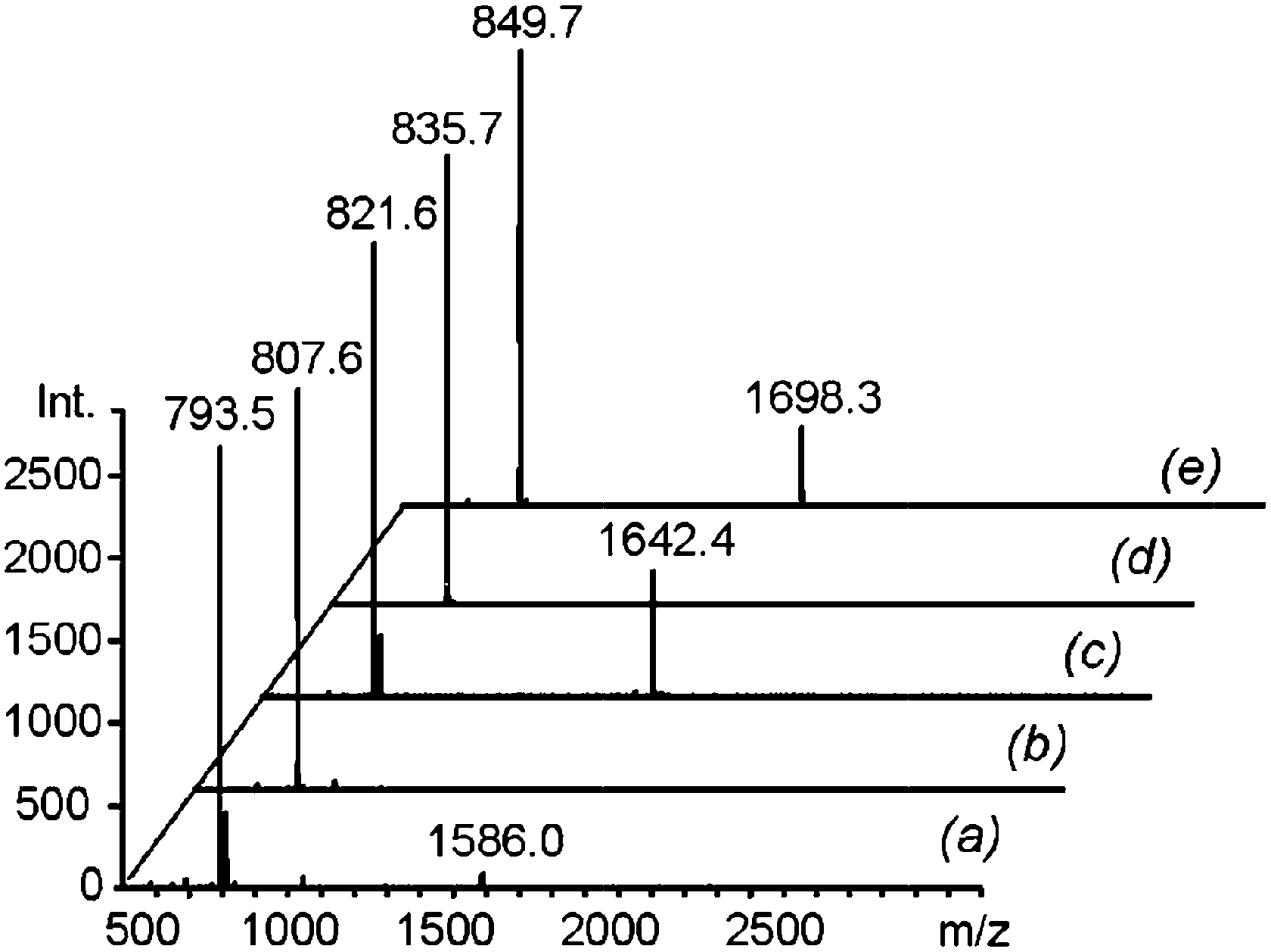
MALDI mass spectra of equimolar mixtures of **1** (2.5 mM) with each of the diamines **3a–e** after equilibration in CD_3_CN ((a) to (e), respectively).

The identities of the macrocycles were revealed by MALDI-TOF analysis of the DLs. The mass spectra showed peaks corresponding to the mass of both the cyclic monomer (**C_1_**) and the cyclic dimer (**C_2_**) in the equilibrated mixtures of **1** with diamines **3a**, **3c** and **3e** (Fig. 9a, c and e, respectively). In the libraries generated with diamines **3b** and **3d**, only **C_1_** was observed (Fig. 9b and d, respectively), consistent with the ^1^H-NMR results. The absence of any peak with a mass corresponding to a linear oligomer is evidence that the concentration used was well below the critical monomer concentration.

**Fig. 10:**
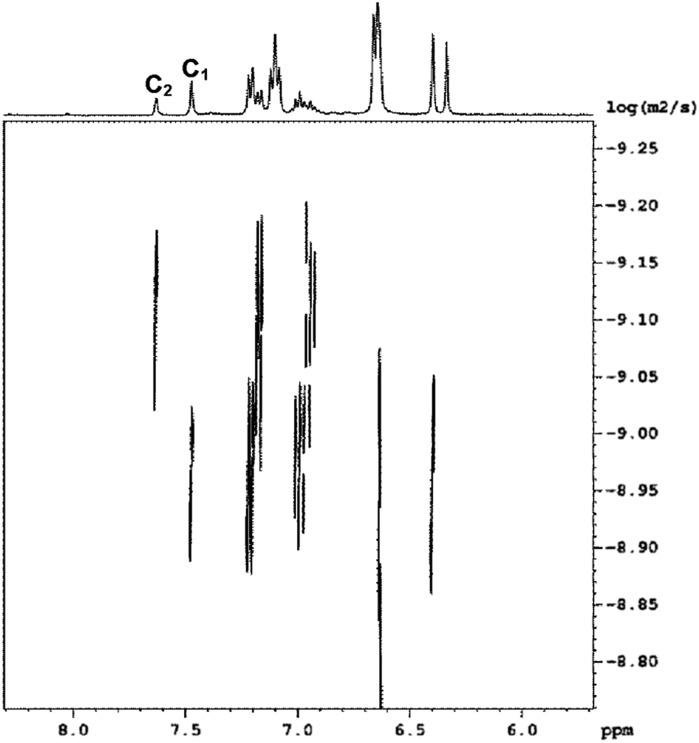
Aromatic region of the DOSY ^1^H NMR spectrum of an equilibrated 1 : 1 mixture of **1** (2.5 mM) and **3c** recorded in CD_3_CN at 298 K.

Diffusion-ordered ^1^H-NMR spectroscopy (DOSY) experiments were used to assign the ^1^H-NMR signals of the macrocycles. DOSY spectra of the equilibrated mixtures of **1** with **3b** or **3d** confirmed the presence of a single compound as all of the signals have the same diffusion coefficient. On the other hand, DOSY spectra of the DL generated from mixtures of **1** and **3a**, **3c** or **3e** exhibited two sets of signals corresponding to two distinct compounds with different diffusion coefficients (Fig. 10 and Table 1).

**Table 1:** Ratios of the diffusion coefficients obtained for the equimolar mixtures of **1** (2.5 mM) and **3a**, **3c** and **3e** equilibrated in CD_3_CN at 298 K. The ratios of molecular weights were calculated using eqn (4) (idealised rod) and eqn (5) (idealised sphere)

	D(**C_1_**)/D(**C_2_**)	Rod	Sphere
		MW_2_/MW_1_	MW_2_/MW_1_
**1** + **3a**	1.40	1.96	2.74
**1** + **3c**	1.31	1.72	2.25
**1** + **3e**	1.37	1.88	2.57

The diffusion coefficient is inversely proportional to the hydrodynamic radius *r*_s_ of the molecule, which is related to the shape and molecular weight: for a rod, *r*_s_ varies as the square root of the molecular weight (eqn (4)); for a sphere, *r*_s_ varies as the cube root of the molecular weight (eqn (5)).4
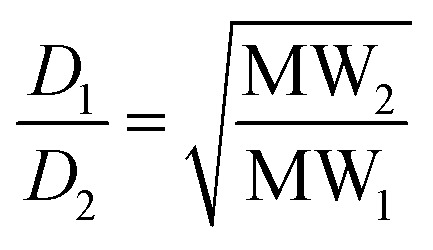

5
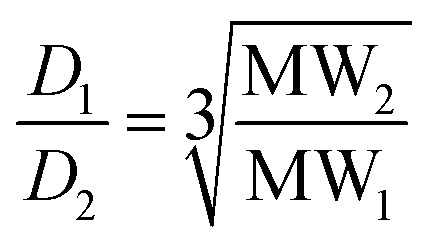



Real molecules have a shape that is intermediate between these idealised extremes, so measurement of the ratio of the diffusion coefficients of two oligomers by DOSY, D_2_/D_1_, provides a method for determining the ratio of the molecular weights, MW_2_/MW_1_.^19^ Table 1 shows the ratio of molecular weights calculated for the ideal rod and sphere models. For all three DLs, the integer that lies between the two values of MW_2_/MW_1_ is 2. Although this result could also be interpreted as a mixture of dimeric and tetrameric oligomers, assignment as a mixture of monomer, **C_1_**, and dimer, **C_2_**, is in agreement with the mass spectrometry results.

**Fig. 11:**
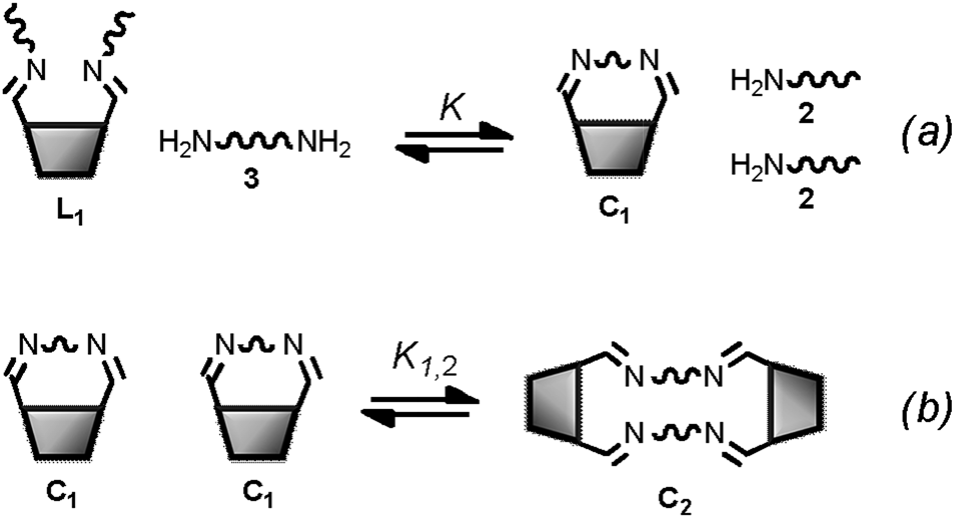
(a) Equilibrium used for determination of the effective molarity of a macrocyclic diimine, **C_1_**. (b) Equilibrium considered for determination of the effective molarity of a macrocyclic tetraimine, **C_2_**.

The monofunctional amine **2** was then added to each DL in order to determine the effective molarities (see ESI for experimental data†). Fig. 11a and b illustrate the equilibria considered for the measurement of the EM of the cyclic monomer and the cyclic dimer, respectively, according to eqn (6) and (7). It is worth noticing that the equilibrium constant *K* defined in eqn (6) differs from the one presented in the more general eqn (2) since *K* is the result of two consecutive equilibria. Displacement of one of the monoamines of **L_1_** by diamine **3** occurs first, followed by the cyclization process (see ESI for details†).6
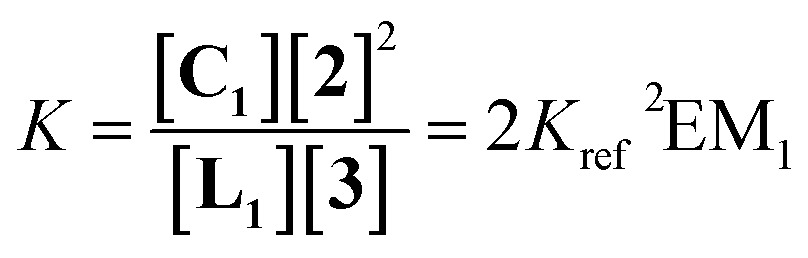

7
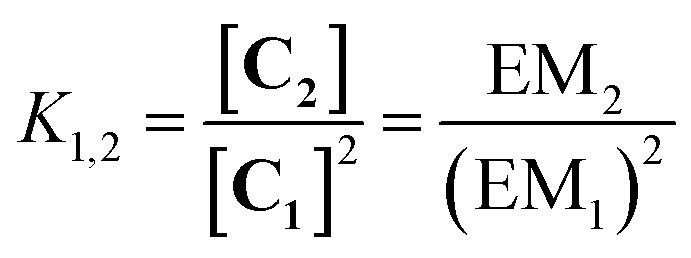



**Fig. 12:**
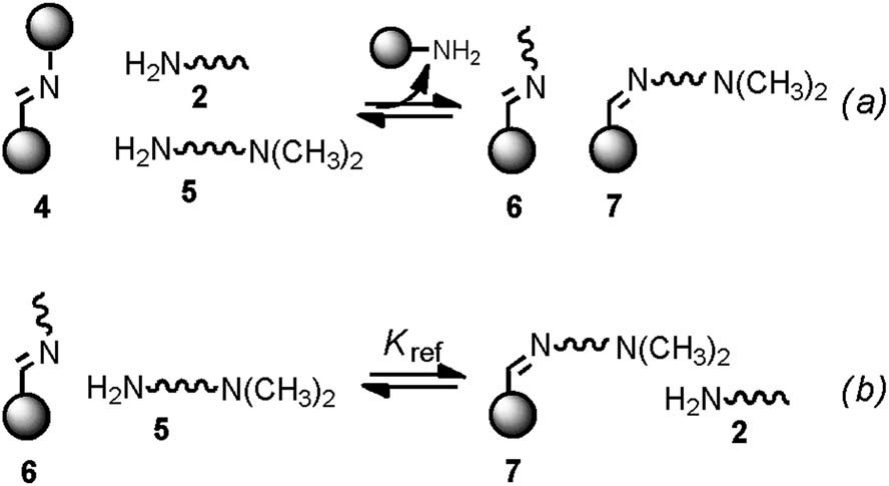
(a) Schematic representation of the generation of dynamic systems from imine **4** (see Fig. 6 for key). (b) Equilibrium used to determine *K*_ref_.

Monofunctional analogues of the diamines **3** (monoamines **5** in Fig. 6b) were used to measure *K*_ref_, the equilibrium constant for the corresponding intermolecular reaction. Fig. 12a shows a schematic representation of the experiment. Measurement of the concentrations of the products allowed determination of *K*_ref_ according to the equilibrium represented in Fig. 12b. Signal overlap in the ^1^H-NMR spectra prevented measurement of *K*_ref_ for amines **5d** and **5e**, but *K*_ref_ is approximately one for **5b** and **5c**, so a value of one was also used for **5d** and **5e**. Table 2 reports the equilibrium constants for the equilibria depicted in Fig. 11a, b and 12b and the values of EM derived from them.

**Table 2:** Equilibrium constants and effective molarities for formation of macrocycles **C_1_** and **C_2_** in CD_3_CN at 298 K

	*K* ^*a*^ (M)	*K*_1,2_ ^*a*^ (M^–1^)	*K*_ref_	EM_1_ (M)	EM_2_ (M)
**3a**	0.025 ± 0.008	110 ± 10	0.5 ± 0.2	0.05	0.3
**3b**	0.9 ± 0.4	—	1.0 ± 0.2	0.5	—
**3c**	0.03 ± 0.02	180 ± 30	1.2 ± 0.2	0.01	0.02
**3d**	3 ± 2	—	1^*b*^	1.5	—
**3e**	0.026 ± 0.008	130 ± 60	1^*b*^	0.01	0.01

^*a*^Errors are quoted at the 95% confidence limit (see Experimental section).

^*b*^Estimated values based on similarity with the previous ones.

**Fig. 13:**
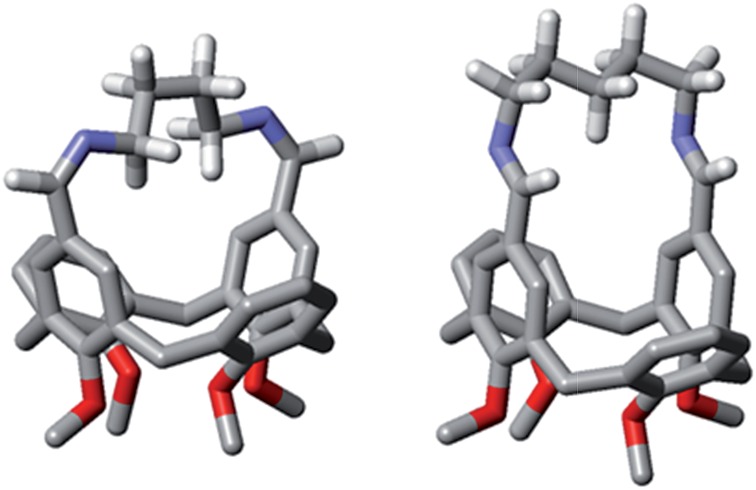
Models of **C_1_c** (left) and **C_1_d** (right) obtained by energy minimisation using macromodel and the MMFFs force field. Only the diamine linker hydrogen atoms are shown for clarity.

**Fig. 14:**
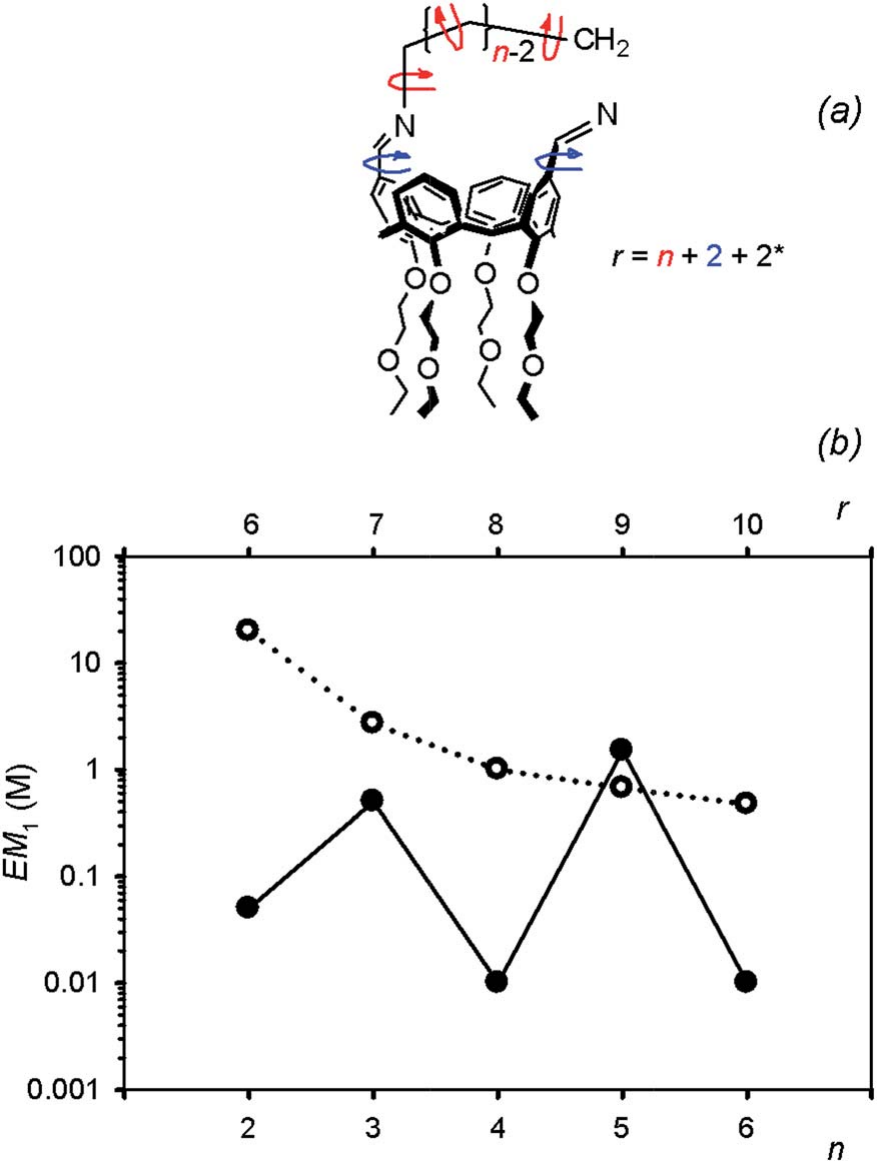
(a) Method used to count rotors in hypothetical linear precursors of **C_1_**; *n* is the number of methylenes between the nitrogen atoms of the diamine. The entropic contribution related to *cone*-calix[4]arene breathing is assimilated into two single bond rotors (see text and ref. 20) (b) plot of EM for cyclic monomers **C_1_** (filled circles) as a function of diamine chain length expressed both as number of methylenes *n* (bottom scale) and number of rotors frozen on cyclization *r* (top scale) as calculated in (a). The corresponding EM values reported in the literature for strain free cyclisation reactions are plotted as empty circles (see ref. 9b and c).

The differences observed in the composition of the DLs for different numbers of methylene units in the diamine chain are reflected in a clear trend in the values of EM. The values of EM_1_ for cyclic diimines with an even number of methylene units in the linker (**C_1_a**, **C_1_c** and **C_1_e**) are significantly lower than the values obtained for **C_1_b** and **C_1_d**, which have an odd number of methylene units. This behaviour cannot be explained in terms of differences in the entropic contribution, as the entropic change associated with the freezing of internal rotors increases uniformly with the length of the linker. There must be a difference in the ring strain in the cyclic diimines, due to differences in conformation. Molecular modeling suggests that linkers containing an odd number of methylenes can adopt a low energy all *anti* conformation, whereas linkers with an even number of methylenes are forced to assume higher energy conformations with *gauche* arrangements (Fig. 13). Interestingly, the value of EM_1_ for **C_1_d** is in reasonable agreement with the value (0.67 M) estimated for a strainless cycle^9*b*^ when the same number of rotors (7 + 2)^20^ of a linear precursor has to be frozen in the ring-closing process (Fig. 14).

## Conclusions

The dynamic libraries presented in this work are minimal libraries of interconverting calixarene-imine macrocycles in CD_3_CN. Characterization of the systems through 1D and 2D NMR spectroscopy and mass spectrometry is possible by working at concentrations well below the critical value, so that only cyclic species are present. It is possible to fine tune the composition of the system by changing the number of methylene units in the aliphatic linkers used. For odd numbers of methylene units, only a monomeric calixarene macrocycle is observed, but for even numbers of methylene units, the dimeric calixarene macrocycle is also populated. The use of monofunctional chain stoppers has allowed us to determine EM values for these systems by introducing linear oligomers into the DLs. For the monomeric calixarene macrocycles, there is an alternating pattern of EM values as the number of methylene units increases: high EM values for odd numbers of methylenes, and low EM values for even numbers of methylenes. This is the first time that this pattern has been observed for large rings and the effect is caused by gauche conformations in the even number chains, which increase the strain energies associated with the cyclisation reaction. In contrast, one of the odd number chains gives a macrocycle which has an EM consistent with a strainless ring. This work represents the first example of the use of dynamic combinatorial chemistry to determine EM in the low concentration domain. The same approach could be extended to supramolecular systems allowing a direct comparison between the behaviour of covalent and non-covalent dynamic libraries.

## Experimental section

### Instruments and methods

^1^H-NMR and ^13^C-NMR spectra were recorded on a 400 MHz spectrometer. Chemical shifts are reported as *δ* values in ppm, all the spectra being internally referenced to the residual proton solvent signal. High-resolution mass spectra (HR-MS) were performed by an Electrospray Ionization Time-Of-Flight Waters LCT spectrometer. MALDI mass spectra were recorded on a MALDI Bruker ReflexIII. FTIR spectra were carried out on a Perkin-Elmer Spectrum 100 spectrometer.

### Materials

All reagents and solvents were used without further purification with the exception of the aniline, which was distilled on NaOH prior to use. Deuterated acetonitrile was dried over activated molecular sieves (4 Å).

### Synthesis of compound **1**

500 mg (0.65 mmol) of 5,17-diformyl-25,26,27,28-tetrakis-(2-ethoxyethoxy)calix[4]arene were dissolved in 600 μl of anhydrous acetonitrile and then aniline (302 mg, 3.25 mmol) was added. The solution was stirred at room temperature and the progress of the reaction was monitored by ^1^H-NMR spectroscopy. After all the aldehyde was consumed, the solvent and the excess of aniline were evaporated under reduced pressure to give the pure product in quantitative yield as a pale yellow wax (597 mg, 0.65 mmol, 100% yield). ^1^H-NMR (400 MHz, CD_3_CN): *δ* 8.28 (s, 2H), 7.44 (s, 4H), 7.34 (t, *J* = 7.5 Hz, 4H), 7.20 (t, *J* = 7.5 Hz, 2H), 7.10 (d, *J* = 7.5 Hz, 4H), 6.69 (d, *J* = 7.5, 4H), 6.59 (t, *J* = 7.5 Hz, 2H), 4.63 (d, *J* = 13.0 Hz, 4H), 4.29 (t, *J* = 5.0 Hz, 4H), 4.12 (t, *J* = 5.0 Hz, 4H), 3.93 (t, *J* = 5.0 Hz, 4H), 3.89 (t, *J* = 5.0 Hz, 4H), 3.61–3.53 (m, 8H), 3.33 (d, *J* = 13.0 Hz, 4H), 2.39 (m, 12H); ^13^C-NMR (100.6 MHz, CD_3_CN): *δ* 161.2, 161.1, 157.1, 153.3, 137.4, 135.4, 131.8, 130.1, 130.0, 129.3, 126.4, 123.4, 121.7, 74.8, 74.8, 70.9, 70.7, 67.0, 66.9, 31.6, 15.7; HRMS (ESI^+^): calcd for C_58_H_66_N_2_O_8_–H^+^ 919.4897; found 919.4922 FT-IR (thin film): *ν*_max_/cm^–1^ 3363, 2923, 1626, 1589, 1451, 1247, 1122.7, 668.

### Synthesis of 4-methoxybenzylideneaniline (**4**)

293 mg (2.15 mmol) of 4-methoxybenzaldehyde and 200 mg (2.15 mmol) of aniline were dissolved in 4 ml of benzene and refluxed in a Dean–Stark apparatus for 4 h. The benzene was then evaporated under reduced pressure and the pure product was obtained in quantitative yield as a pale yellow solid (454 mg, 2.15 mmol, 100% yield). ^1^H-NMR (400 MHz, CD_3_CN): *δ* 8.48 (s, 1H), 7.89 (d, *J* = 9.0 Hz, 2H), 7.42 (t, *J* = 9.0 Hz, 2H), 7.26–7.21 (m, 3H), 7.06 (d, *J* = 9.0 Hz), 3.88 (s, 3H); ^13^C-NMR (100.6 MHz, CD_3_CN): *δ* 163.4, 160.8, 153.4, 131.4, 130.4, 130.2, 126.5, 121.8, 115.2, 56.2; HRMS (ESI^+^): calcd for C_14_H_13_NO–H^+^ 212.1075; found 212.1076; FT-IR (thin film): *ν*_max_/cm^–1^ 2258, 2923, 1945, 1605, 1512, 1253, 1164, 1030, 835.

### General procedure for the generation of the dynamic libraries

Stock solutions of the diimine **1** (15 mM), of hexylamine **2** (150 mM) and of each diamine **3** (100 mM) were prepared in deuterated acetonitrile. The aliquots of these solutions were loaded into NMR tubes and then CD_3_CN was added in order to obtain 600 μL of a 1 : 2 : 1 mixture of the substrates (2.5 mM of **1**). The system was then left to equilibrate at 25 °C. After 1–4 days, there was no further change in the ^1^H-NMR spectrum showing that the system had reached equilibrium.

### Determination of equilibrium constants

The equilibrium constants were determined by using the concentrations obtained by integration of the ^1^H-NMR signals, using the signal of the methyl group of the *n*-hexyl chain as an internal reference. The errors in *K*_ref_ were calculated by considering a 5% error in the integrals. Errors in *K* and *K*_1,2_ are quoted as twice the standard deviation of the values obtained from repeating the experiment twice at different concentrations of hexylamine (5 mM, 10 mM and 20 mM).
