# Supplementary material for: Applications of dynamic combinatorial chemistry for the determination of effective molarity
Source: Chem Sci. 2014 Sep 29;6(1):144–51. doi: 10.1039/c4sc02347a (PMC5424495; doi:10.1039/c4sc02347a)

Electronic Supplementary Material (ESI) for Chemical Science.
This journal is © The Royal Society of Chemistry 2014

Supporting Information for

Applications of Dynamic Combinatorial Chemistry for the Determination of Effective Molarity

Maria Ciaccia,[a] Irene Tosi,[b] Laura Baldini,[b] Roberta Cacciapaglia,[a] Luigi Mandolini,[a] Stefano Di Stefano[a] and Christopher A. Hunter*[c]*

[a] Dipartimento di Chimica, Sapienza Università di Roma and Istituto CNR di Metodologie Chimiche (IMC-CNR), Sezione Meccanismi di Reazione, c/o Dipartimento di Chimica, Sapienza Università di Roma, P.le A. Moro, 5 I-00185 Rome, Italy.

[b] Dipartimento di Chimica, Università di Parma, Parco Area delle Scienze 17/A, 43124, Parma, Italy.

[c] Department of Chemistry, University of Sheffield, Sheffield, S3 7HF, UK.

INDEX

Derivation of eqn (3) of the main text (ESI3)

Derivation of eqn (6) of the main text (ESI3)

Derivation of eqn (7) of the main text (ESI5)

^{1}H-NMR spectra of the equilibrated mixtures **1** + **2** + **3a-e** in CD_3CN (ESI6)

Determination of the equilibrium constants K and $K_{1,2}$ (ESI7)

Assignment of ^{1}H-NMR signals for the determination of K_{ref} (ESI8)

^{1}H-NMR spectrum of compound **1** (ESI10)

^{13}C-NMR spectrum of compound **1** (ESI11)

^{1}H-NMR spectrum of compound **4** (ESI12)

^{13}C-NMR spectrum of compound **4** (ESI13)

Derivation of eqn (3) of the main text

Since

$$K_{m,n} = \frac{(\mathcal{EM}_n)^m}{(\mathcal{EM}_m)^n}$$

and

$$\mathcal{EM}_n = \frac{1}{\sigma_n}\mathrm{EM}_n$$

then

$$K_{m,n} = \frac{(\mathrm{EM}_n)^m}{(\mathrm{EM}_m)^n} \times \frac{(\sigma_m)^n}{(\sigma_n)^m}$$

See note 12 in the main text.

Derivation of eqn (6) of the main text

The reaction depicted below was used to determine K_{ref}:

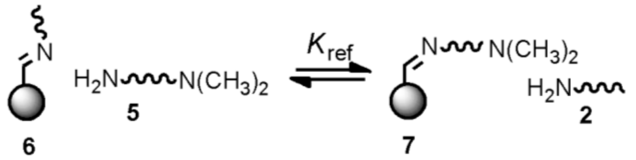

Fig. S1 (top) shows the equilibrium considered to determine EM_1. The formation of the cyclic monomer $\mathbf{C_1}$ from the linear end-capped precursor $\mathbf{L_1}$ is the result of two consecutive equilibria (Fig. S1 bottom). The first step (a) is the nucleophilic attack of the diamine **3** on $\mathbf{L_1}$. Since both reagents are bifunctional and the reacting groups on each substrate are equivalent to each other, the formation of the product can occur in four degenerate ways, whereas the products can go back to the reagents in just one way. The equilibrium constant K_{a} (eqn (S1)) of the process is then equal to the product of the statistical factor 4 and the K_{ref} associated with the intermolecular process.

The second equilibrium (Fig. S1b) is the cyclization process. The reagent can undergo the cyclization in just one way, whereas the product $\mathbf{C_1}$ can react with **2** in two degenerate ways to form the reagents again. For this reason, the statistical factor associated with the process is ½. The

equilibrium constant of the cyclization K_b (eqn (S2) is then the product of the statistical factor ½, the K_{ref} and the effective molarity EM_1. K_b can be assimilated to K_n presented in eqn. (2) in the main text when n = 1. The statistical factor also corresponds to the reciprocal of the symmetry number σ_1 of the monomer $\mathbf{C_1}$.

The equilibrium constant of the whole process K is then the product of K_a and K_b (eqn (S3)), corresponding to eqn (6) in the main text.

$$K_a = 4K_{\text{ref}} \tag{S1}$$

$$K_b = \frac{1}{2}K_{\text{ref}}\text{EM}_1 \tag{S2}$$

$$K = \frac{[\mathbf{C_1}]\,[\mathbf{2}]^2}{[\mathbf{L_1}]\,[\mathbf{3}]} = K_\text{a} \times K_\text{b} = 4K_{\text{ref}} \times \frac{1}{2}K_{\text{ref}}\text{EM}_1 = 2K_{\text{ref}}^2\,\text{EM}_1 \tag{S3}$$

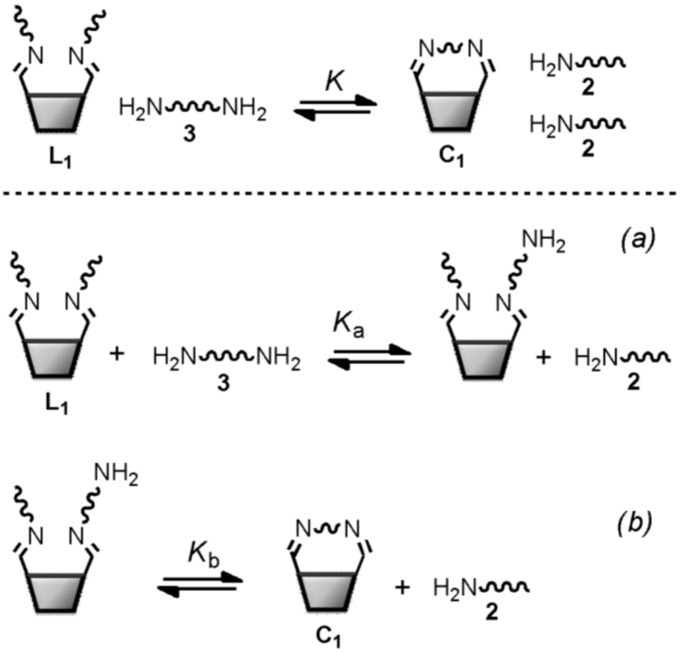

Fig. S1 Top: Equilibrium between the end-capped linear species $\mathbf{L_1}$ and the cyclic monomer $\mathbf{C_1}$ used to determine EM_1. Bottom: Equilibria involved in the process depicted on top: nucleophilic attack of the diamine **3** on $\mathbf{L_1}$ (a) followed by the cyclization process (b).

Derivation of eqn (7) of the main text

The equilibrium depicted in Fig. S2 was used to determine the effective molarity of **C_2**. Eqn (S4) corresponds to eqn (7) in the main text when the symmetry number of the two cycles are explicitely expressed (*cf* also with eqn (3) in the main text with m = 1 and n = 2). σ is 2 for **C_1**, since it can be opened in two degenerate ways to form a linear precursor, and 4 for **C_2** since there are four equivalent ways to re-open the cycle (see ref. 14 in the main text).

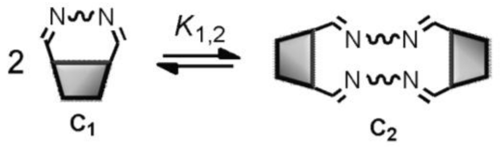

Fig. S2 Equilibrium between the cyclic monomer **C_1** and the dimer **C_2** used to determine of EM_2.

$$K_{1,2} = \frac{[\mathbf{C_2}]}{[\mathbf{C_1}]^2} = \frac{EM_2}{(EM_1)^2} \times \frac{(2)^2}{4} = \frac{EM_2}{(EM_1)^2} \qquad (S4)$$

^{1}H-NMR spectra of the equilibrated mixtures 1 + 2 + 3a-e in CD_3CN

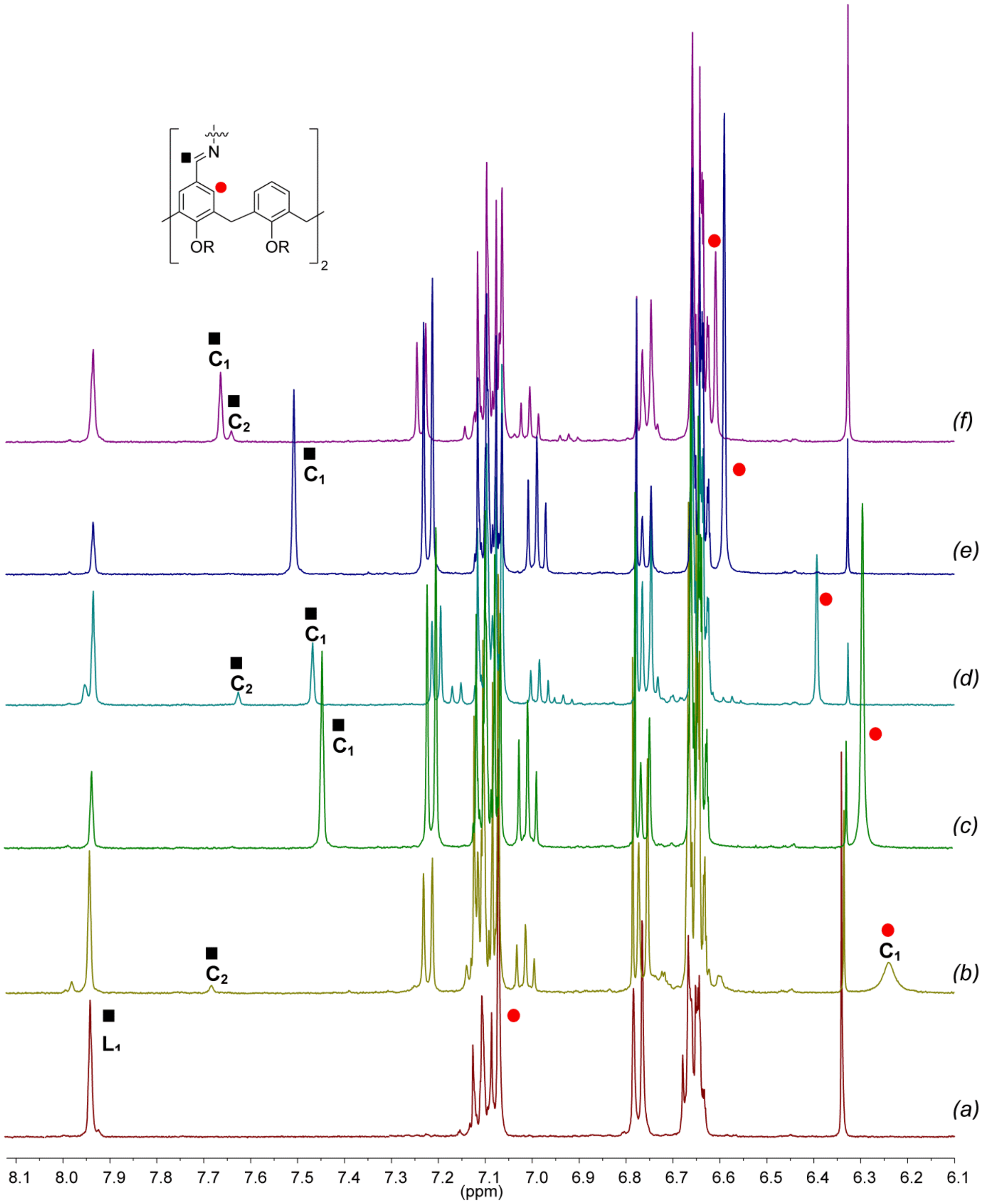

Fig S3 Aromatic region of the ^{1}H-NMR spectra of $\mathbf{L_1}$ (a) and of equilibrated 1:2:1 mixtures of **1** (2.5 mM), **2** and each of the diamines **3a-e** (b-f) recorded in CD_3CN at 298 °K. The black squares are used to mark the imine signals and the red circles are used to mark the aromatic protons on the phenyl ring carrying the imine moiety as schematically depicted at the top of the figure.

Determination of the equilibrium constants *K* and $K_{1,2}$

Table S1 Concentrations of the species at the equilibrium in a set of experiment carried out on the DL generated from **1** and **3a** (2.5 mM in CD_3CN, 298 °K) at increasing concentrations of **2** and corresponding equilibrium constants.

$[\mathbf{2}]_o$ (mM)	$[\mathbf{2}]_e$ (mM)	$[\mathbf{L_1}]_e$ (mM)	$[\mathbf{3}]_e$ (mM)	$[\mathbf{C_1}]_e$ (mM)	$[\mathbf{C_2}]_e$ (mM)	*K* (M)	$K_{1,2}$ (M^{-1})
5.0	4.0	0.5	1.2	1.0	0.1	0.027	100
10.0	8.2	0.9	1.7	0.7	0.05	0.031	102
20.0	17.2	1.4	2.2	0.3	-	0.029	-

The concentrations of the imine derivatives **L_1**, **C_1** and **C_2** were obtained by direct integration of the corresponding signals in the ^{1}H-NMR spectra recorded at the equilibrium. The concentrations of the amines **2** and **3** were obtained by the mass balance equations (eqns S5 and S6).

$$[\mathbf{2}]_e = [\mathbf{2}]_0 - 2[\mathbf{L_1}]_e \quad \text{(S5)}$$

$$[\mathbf{3}]_e = [\mathbf{3}]_0 - [\mathbf{C_1}]_e - 2[\mathbf{C_2}]_e \quad \text{(S6)}$$

Assignment of ¹H-NMR signals for the determination of K_{ref}

The values of K_{ref} reported in Table 2 of the main text were obtained by integration of the signals in the ¹H-NMR spectra of the equilibrated mixtures of compounds **4**, **2** and **5**.

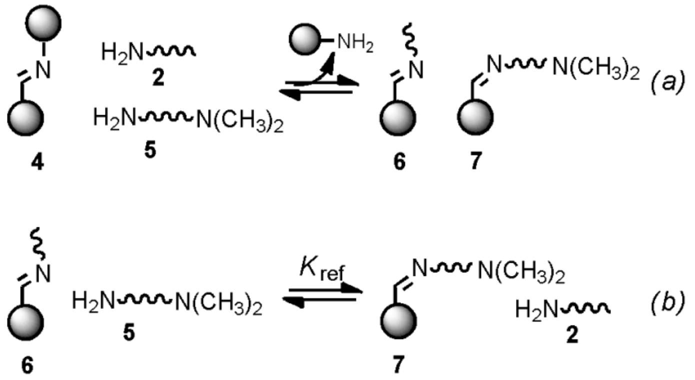

The regions of the ¹H-NMR spectra used for the assignment of the species are reported in Fig. S4, S5 and S6 for amines **5a**, **5b** and **5c**, respectively.

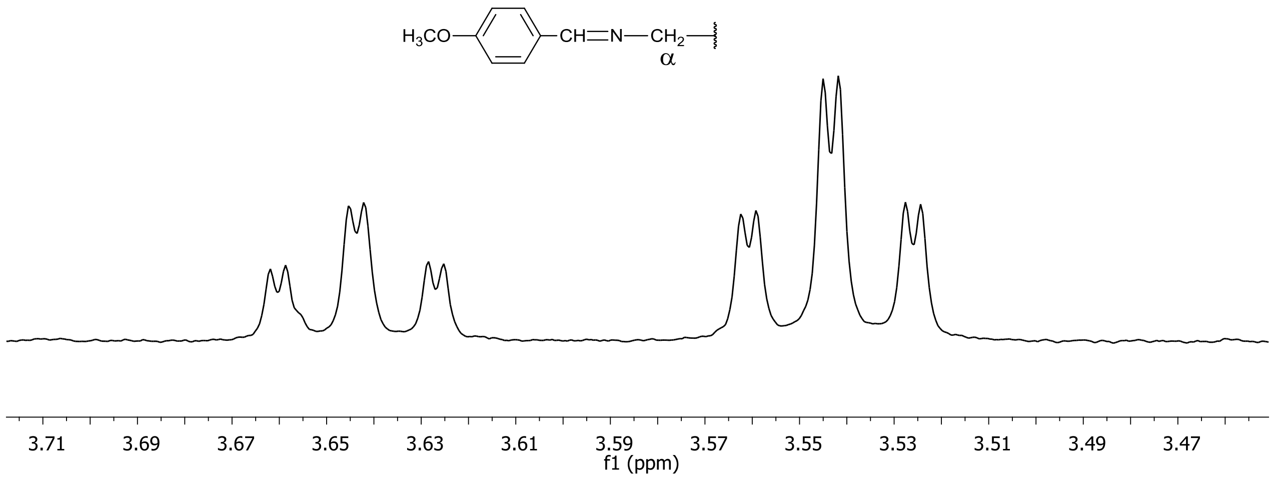

Fig. S4 Partial ¹H-NMR spectrum of the equilibrated 1:1:1 mixture of **4**, **2** and **5a** (5 mM, CD_3CN, T = 298 °K). The signal at δ = 3.64 ppm corresponds to the methylene in α to the nitrogen in imine **7a**; the signal at δ = 3.54 ppm corresponds to the methylene in α to the nitrogen in imine **6**.

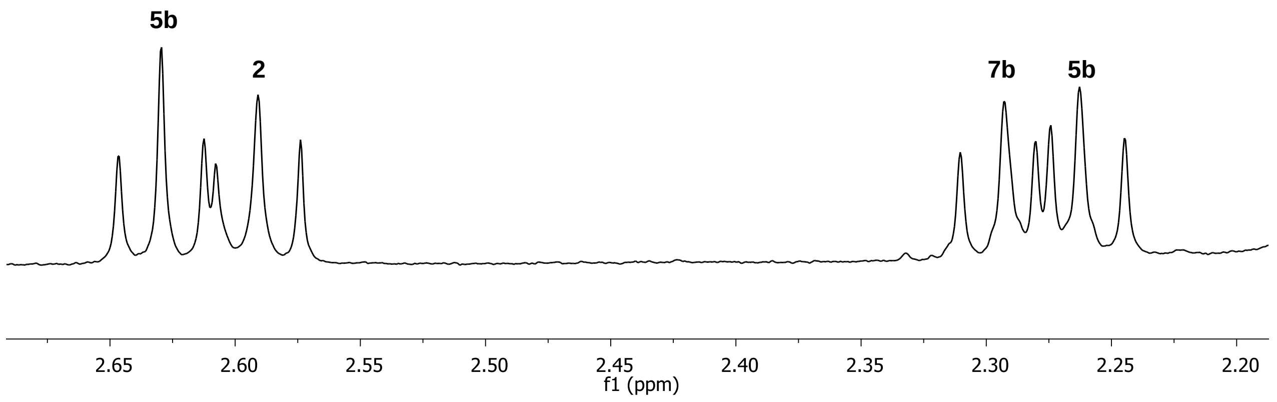

Fig. S5 Partial ^{1}H-NMR spectrum of the equilibrated 1:1:1 mixture of **4**, **2** and **5b** (5 mM, CD_3CN, T = 298 °K).

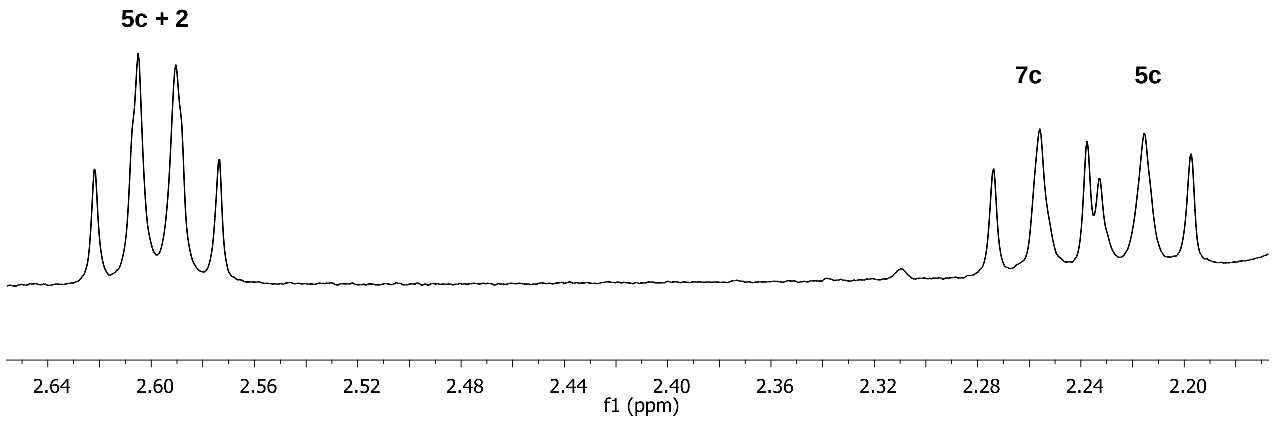

Fig. S6 Partial ^{1}H-NMR spectrum of the equilibrated 1:1:1 mixture of **4**, **2** and **5c** (5 mM, CD_3CN, T = 298 °K).

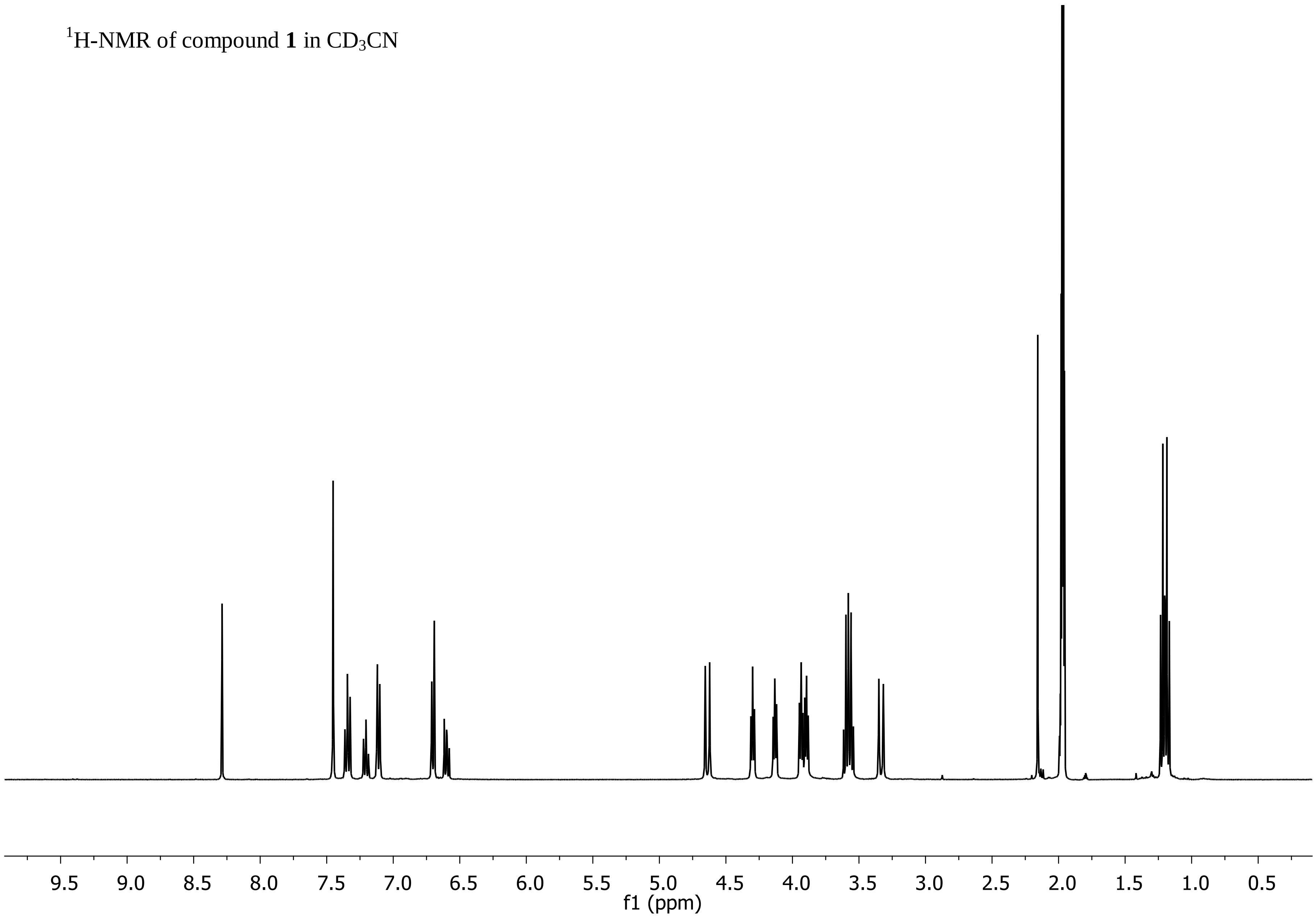
1H-NMR of compound 1 in CD3CN
9.5 9.0 8.5 8.0 7.5 7.0 6.5 6.0 5.5 5.0 4.5 4.0 3.5 3.0 2.5 2.0 1.5 1.0 0.5
f1 (ppm)

^{13}C-NMR of compound **1** in CD_3CN

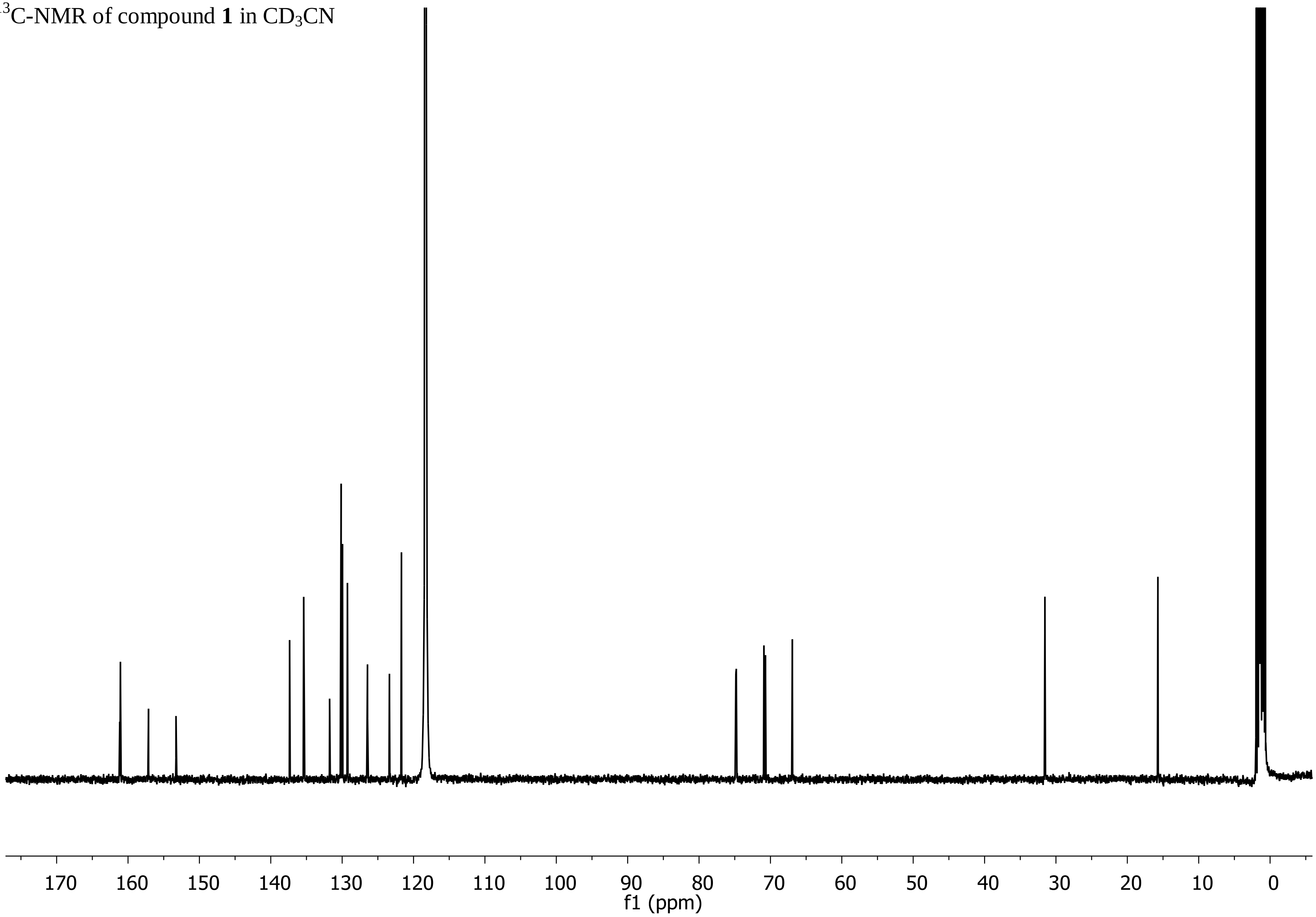

^{1}H-NMR of compound **4** in CD_3CN

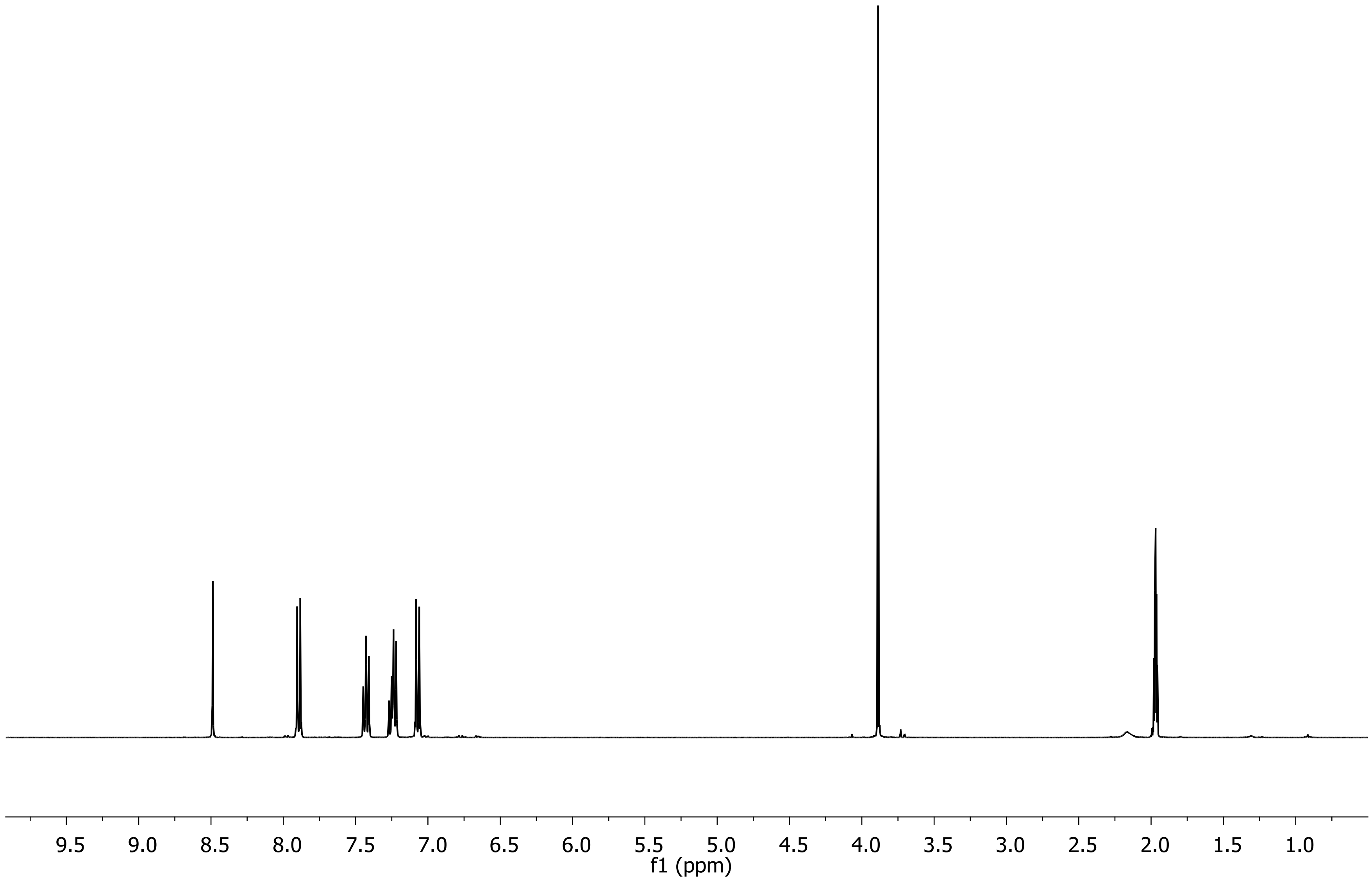

^{13}C-NMR of compound **4** in CD_3CN

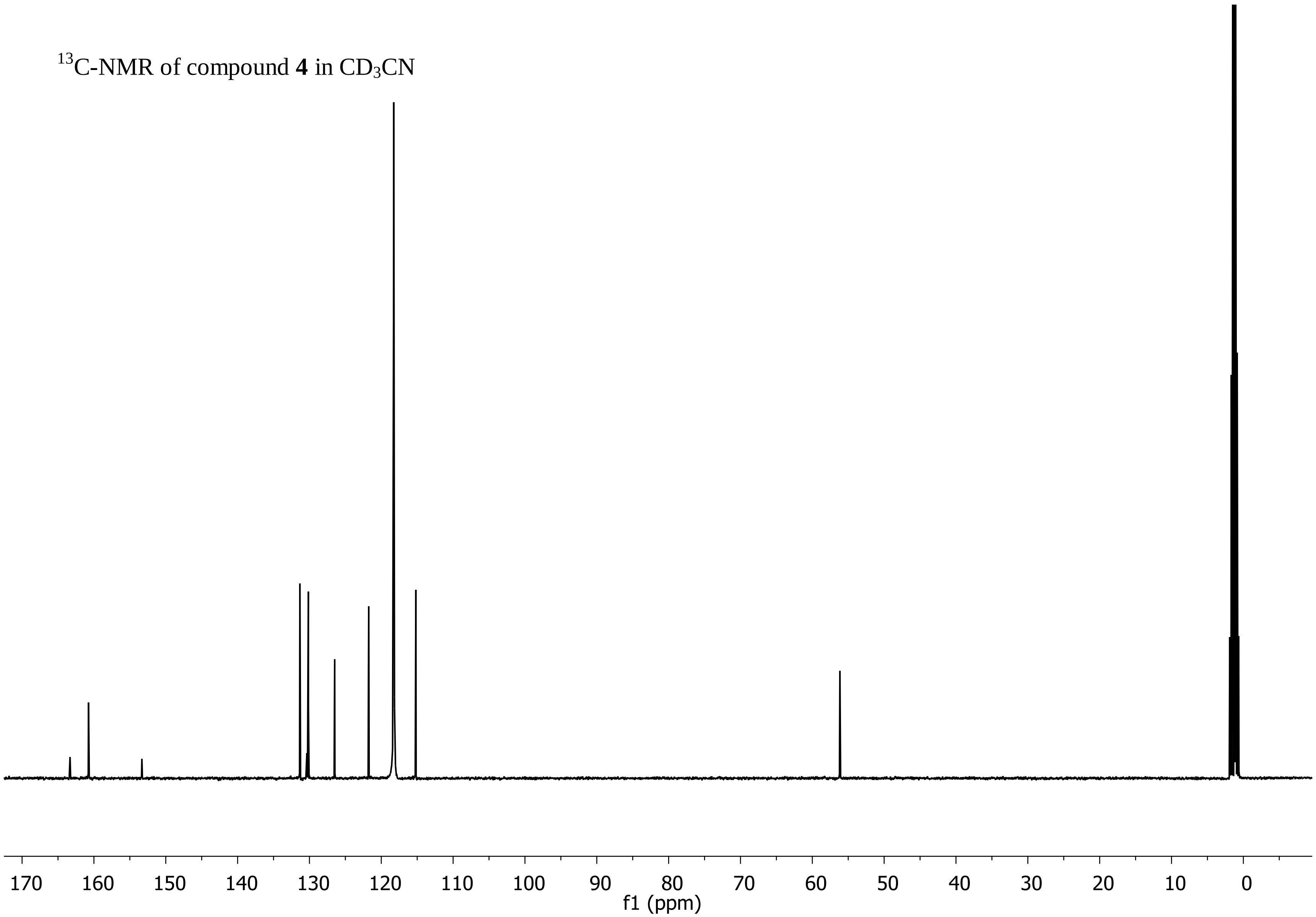

Supplement: Supplementary file 1 [file SC-006-C4SC02347A-s001.pdf]
